# Anti-seizure effects of JNJ-54175446 in the intra-amygdala kainic acid model of drug-resistant temporal lobe epilepsy in mice

**DOI:** 10.3389/fphar.2023.1308478

**Published:** 2024-01-08

**Authors:** Omar Mamad, Mona Heiland, Andreas U. Lindner, Thomas D. M. Hill, Ronan M. Ronroy, Kilian Rentrup, Amaya Sanz-Rodriguez, Elena Langa, Janosch P. Heller, Oscar Moreno, Jordi Llop, Anindya Bhattacharya, James A. Palmer, Marc Ceusters, Tobias Engel, David C. Henshall

**Affiliations:** ^1^ Department of Physiology and Medical Physics, RCSI University of Medicine and Health Sciences, Dublin, Ireland; ^2^ FutureNeuro SFI Research Centre, RCSI University of Medicine and Health Sciences, Dublin, Ireland; ^3^ Division of Population Health Sciences, RCSI University of Medicine and Health Sciences, Dublin, Ireland; ^4^ School of Biotechnology, Dublin City University, Dublin, Ireland; ^5^ CIC biomaGUNE, Basque Research and Technology Alliance (BRTA), San Sebastián, Spain; ^6^ Neuroscience, Janssen Pharmaceutical Research and Development, LLC, San Diego, CA, United States; ^7^ Neuroscience, Janssen Pharmaceutical Research and Development, Janssen Pharmaceutica NV, Beerse, Belgium

**Keywords:** anti-seizure medicines, inflammation, interleukin 1β, drug-resistant epilepsy, hippocampal sclerosis

## Abstract

There remains a need for new drug targets for treatment-resistant temporal lobe epilepsy. The ATP-gated P2X7 receptor coordinates neuroinflammatory responses to tissue injury. Previous studies in mice reported that the P2X7 receptor antagonist JNJ-47965567 suppressed spontaneous seizures in the intraamygdala kainic acid model of epilepsy and reduced attendant gliosis in the hippocampus. The drug-resistance profile of this model is not fully characterised, however, and newer P2X7 receptor antagonists with superior pharmacokinetic profiles have recently entered clinical trials. Using telemetry-based continuous EEG recordings in mice, we demonstrate that spontaneous recurrent seizures in the intraamygdala kainic acid model are refractory to the common anti-seizure medicine levetiracetam. In contrast, once-daily dosing of JNJ-54175446 (30 mg/kg, intraperitoneal) resulted in a significant reduction in spontaneous recurrent seizures which lasted several days after the end of drug administration. Using a combination of immunohistochemistry and *ex vivo* radiotracer assay, we find that JNJ-54175446-treated mice at the end of recordings display a reduction in astrogliosis and altered microglia process morphology within the ipsilateral CA3 subfield of the hippocampus, but no difference in P2X7 receptor surface expression. The present study extends the characterisation of the drug-resistance profile of the intraamygdala kainic acid model in mice and provides further evidence that targeting the P2X7 receptor may have therapeutic applications in the treatment of temporal lobe epilepsy.

## Introduction

Epilepsy is characterised by recurring spontaneous seizures and is among the most common and disabling brain diseases (Devinsky et al., 2018). Frontline treatment for epilepsy is based on small molecule drugs that target ion channels and neurotransmitter systems, including levetiracetam and other synaptic vesicle glycoprotein 2A (SV2A)-modulating compounds (Kwan et al., 2011; Loscher et al., 2016). Unfortunately, about one-third of patients fail to achieve seizure control and current anti-seizure medicines do not modify the underlying pathophysiology (Simonato et al., 2012; Loscher et al., 2020). Accordingly, new treatments are urgently required for treatment-resistant epilepsy, including patients with temporal lobe epilepsy (TLE) (Loscher et al., 2013).

Molecular and cellular analyses of resected brain tissue from patients, as well as from experimental models, has identified elevated inflammatory signalling in TLE (Vezzani et al., 2011; Aronica et al., 2017; Vezzani et al., 2019). This includes raised levels of cytokines and activation of capase-1, which catalyses maturation of interleukin 1β (IL-1β), a potent neuromodulator and activator of gliosis (Allan et al., 2005). The caspase-1/IL-1β system lies downstream of the surface expressed ATP-gated P2X7 receptor (Beamer et al., 2017; Dutta et al., 2022). In the brain, the P2X7 receptor is expressed by resident and activated microglia and has been reported to be present on other cell types, particular after injury (Sim et al., 2004; Anderson and Nedergaard, 2006; Kaczmarek-Hajek et al., 2018). Activation of the receptor coordinates neuroinflammatory responses that initially resolve tissue injury (Roth et al., 2014; Di Nunzio et al., 2021), but prolonged or excessive activation may contribute to pathological states of hyper-excitability (Aronica et al., 2017; Vezzani et al., 2019). Over-expression of the P2X7 receptor is found within the seizure focus in experimental and human epilepsy (Rappold et al., 2006; Jimenez-Pacheco et al., 2016; Wei et al., 2016), an observation recently extended by the use of P2X7 receptor radiotracers (Mikkelsen et al., 2023; Morgan et al., 2023). Antagonism or genetic disruption of the P2X7 receptor has been reported to reduce evoked seizures in rodents (Beamer et al., 2017). Recently, treatment of mice with the P2X7 receptor antagonist JNJ-47965567 was found to reduce the frequency of spontaneous seizures in a mouse TLE model (Jimenez-Pacheco et al., 2016). Notably, seizure suppression continued beyond the period of active dosing and was associated with reduced gliosis in the hippocampus (Jimenez-Pacheco et al., 2016), suggesting a potential disease-modifying effect.

The intraamygdala kainic acid (IAKA) model in mice has become increasingly adopted for studies on TLE (Henshall, 2017; Rusina et al., 2021; West et al., 2022). The status epilepticus triggered by IAKA causes damage to the ipsilateral hippocampus and within a few days the emergence of recurrent spontaneous seizures (Mouri et al., 2008). The model reflects aspects of the neuropathology and gene expression landscape of human TLE (Conte et al., 2020), has been adapted to various background strains and used to demonstrate effects of multiple genes, including the P2X7 receptor (Engel et al., 2012; Beamer et al., 2022). Recent studies show the epileptic seizures in the IAKA model are refractory to various conventional anti-seizure medicines (Welzel et al., 2020; West et al., 2022). The profile of levetiracetam in the model is unknown.

JNJ-54175446 is a recently discovered potent, selective and brain-penetrant P2X7 receptor antagonist which has entered clinical trials for other CNS disorders (Letavic et al., 2017; Recourt et al., 2020; Recourt et al., 2023). Here, we extend the characterization of the drug-resistance profile of the IAKA model by testing the effects of levetiracetam. We then assessed the effects of JNJ-54175446 on spontaneous seizures and analyzed gliosis in the brains of mice previously treated with JNJ-54175446. We report that the model is refractory to levetiracetam. In contrast, JNJ-54175446 resulted in a modest reduction in spontaneous seizures which was most apparent following the period of active dosing.

## Materials and methods

### Animals

All experimental procedures involving animals were carried out in accordance with the European Communities Council Directive (2010/63/EU). Procedures using adult male C57BL/6JOlaHsd mice (25–30 g, Harlan) were approved by the Research Ethics Committee (REC 1587) of the RCSI University of Medicine and Health Sciences, under license from the Ireland Health Products Regulatory Authority (AE19127/P057). Animals were housed on a 12 h light-dark cycle under controlled conditions (temperature: 20°C–25°C; humidity: 40%–60%). Food and water were available *ad libitum*.

### Intraamygdala kainic acid (IAKA) model

Epilepsy was induced using the IAKA model technique (Jimenez-Pacheco et al., 2016). Briefly, male C57BL/6JOlaHsd mice (weight: 28–30 g; age 10 weeks) were anaesthetised with isoflurane (5% induction, 2% maintenance) and placed in a mouse-adapted stereotaxic frame. An EEG transmitter unit (model HDX-02; Data Systems International (DSI), MN, United States of America) was implanted under the skin in a subcutaneous pocket along the dorsal flank of the mouse. The EEG signal was recorded from skull-fixed screws over the dorsal hippocampi. A guide cannula was placed above the right basolateral amygdala (coordinates from adjusted Bregma: anterior-posterior (A/P) = −0.95 mm; lateral (L) = −2.85 mm). After 48 h recovery, all animals received an intraamygdala microinjection of KA (0.3 µg in 0.2 µL volume). Status epilepticus developed and was recorded using the surface EEG. After 40 min, all mice received an intraperitoneal (IP) injection of lorazepam (8 mg/kg; Pfizer) in order to reduce morbidity and mortality. Mice were returned to their home cages and stayed under climate-controlled conditions and video monitoring combined with telemetry. EEG was recorded from individually-housed, freely-moving mice for 24 h/day up to 5 weeks after KA injection. After the recording period, animals were transcardially perfused with phosphate-buffered saline (PBS) and whole brains were collected and directly flash-frozen.

### EEG and seizure analysis

The number and duration of spontaneous seizures were determined by individually reviewing EEG records, as described previously using LabChart 8 Reader (AD Instruments, Oxford, UK) (Jimenez-Pacheco et al., 2016). Spontaneous seizures were defined as high-frequency (>5 Hz), high-amplitude (more than two times baseline) polyspike discharges of ≥10 s duration that were present on both EEG channels. Seizure termination was defined as a return of EEG amplitude and frequency to baseline values with or without postictal amplitude suppression.

### Drug administration

Due to solubility and oral bioavailability, delivery of levetiracetam to mice was achieved by dissolving in drinking water, guided by previous studies (Klitgaard et al., 1998; Russo et al., 2010). The ED_50_ (i.e., the dose protecting 50% of the animals) for levetiracetam in the pentylenetetrazol kindling test in mice is 36 mg/kg intraperitoneal (IP) and is 7 mg/kg (IP) in the corneal electroshock kindling the ED_50_ in mice. In rats, levetiracetam reduces pilocarpine-induced seizures at an ED_50_ of 17 mg/kg (IP) and protects against kainic acid seizures at an ED_50_ of 54 mg/kg (IP) (Klitgaard et al., 1998). Based on this, we aimed to reach dosing of ∼80 mg/kg per day, by dissolving 200 mg of levetiracetam in 300 mL of drinking water. Drinking water bottles were covered to exclude light and prepared freshly twice a week. We confirmed this achieved suitable dosing by comparing plasma and brain levels of levetiracetam in mice after consuming the drinking water to the levels achieved following an acute IP injection. Blood samples for plasma analysis of levetiracetam were obtained via submandibular blood draws, as previously described (Brennan et al., 2020). The concentration of levetiracetam in samples was determined by LC-MS/MS.

JNJ-54175446 was dissolved in DMSO (10%) and PEG400 (90%). During the original characterization of JNJ-54175446, the EC_50_ for P2X7 receptor occupancy was a plasma concentration of 105 ng/mL (Letavic et al., 2017). Above 80% receptor occupancy occurs above 1000 ng/mL. An oral administration of 10 mg/kg JNJ-54175446 resulted in >80% receptor occupancy lasting 24 h (Letavic et al., 2017). To determine plasma and brain levels, a set of control and IAKA mice (2 week time-point) received a once daily IP injection of JNJ-54175446. The concentration of JNJ-54175446 was determined by LC-MS/MS. For dosing during epilepsy studies, mice were injected IP at a dose of 30 mg/kg once per day (Letavic et al., 2017) for five consecutive days (n = 8/group). To improve handling and reduce stress and potential displacement of EEG electrodes/telemetry, vehicle and JNJ-54175446-treated mice were briefly and lightly anaesthetised during each IP injection with isoflurane.

### Histology

Whole brains were sectioned (12 µm coronal) on a cryostat (CM 1900, Leica), and mounted on glass slides. Slide-mounted sections were fixed using 4% paraformaldehyde (PFA), then permeabilized and blocked in bovine serum albumin followed by incubation with antibodies against glial fibrillary acidic protein (GFAP) (1:400; Sigma), and ionized calcium binding adaptor molecule 1 (IBA1) (1:400; GeneTex) overnight at 4°C. Sections were washed and then incubated with rabbit polyclonal secondary antibodies coupled to AlexaFluor 488 or AlexaFluor 568 (Invitrogen). To confirm specificity, additional sections were incubated without the primary antibody. Nuclei were labelled by staining with DAPI (4′,6′-diamidino-2-phenylindole dihydrochloride; Vector Laboratories), and sections were examined using a Leica DM4000 epifluorescence microscope. Counts of GFAP+ and IBA1+ cells were performed blind to treatment. One dorsal (−2.8 AP) and one ventral (−2.2 AP) representative region within each hippocampal subfield was selected, and cells were counted under ×20 lens magnification. Representative images of the GFAP/IBA1 staining were taken using the same exposure time without knowledge of the treatment group. We performed an additional analysis of the microglia in the CA3 subfield, by assessing the number and length of processes, as described (Young and Morrison, 2018).

### Radiochemistry and *in vitro* autoradiography

Visualisation of P2X7 receptor binding in the brain was performed by autoradiography. ^18^F-JNJ-64413739 synthesis was performed using a TRACERlab FX_FN_ synthesis module (GE Healthcare) following a previously described method with minor modifications (Morgan et al., 2023). In brief, [^18^F]F^−^ was generated in a Cyclone 18/9 cyclotron (IBA) by proton irradiation of ^18^O-enriched water via the ^18^O (p,n)^18^F nuclear reaction, and trapped on a preconditioned Sep-Pak Accell Plus QMA Light cartridge (Waters). The trapped [^18^F]F^−^ was eluted with a solution of Kryptofix K_2.2.2_/K_2_CO_3_ in a mixture of acetonitrile/water (2:1, 1.5 mL). After complete elimination of the solvent by azeotropic evaporation, a solution containing the precursor (JNJ-64410047, 4 mg) in DMSO (0.7 mL) was added, and the mixture was heated at 120°C for 15 min. After cooling to room temperature, the mixture was purified by high-performance liquid chromatography (HPLC) using a Phenomenex Luna C18 (250 mm) column as the stationary phase and water (0.1% trifluoroacetic acid [TFA])/acetonitrile (65/35, vol/vol) as the mobile phase (flow rate = 4 mL/min). The desired fraction (retention time = 18.0 min) was collected, diluted with a sodium ascorbate solution, and reformulated using a C-18 light cartridge (Sep-Pak Plus, Waters). The resulting ethanol solution (1 mL) was directly used for the *in vitro* studies. Chemical and radiochemical purity were determined by radio-HPLC, and identity of the desired tracer was confirmed by co-elution with reference standard. An Agilent Eclipse XBD-C18 (4.6 × 150 mm, 5 μm) was used as the stationary phase and water (0.1% TFA)/acetonitrile (70/30, vol/vol) as the mobile phase at a flow rate of 1 mL/min (retention time = 12.5 min). Decay-corrected radiochemical yield was 6.9% ± 0.5% (total synthesis time: 90 min). Radiochemical purity was >99% and a molar activity of 47.3 GBq/μmol was obtained at the end of the synthesis.

Coronal brain slices (12 μm) were thawed and pre-incubated for 15 min with Tris-HCl buffer (50 mM, pH 7.4, supplemented with 1 mM MgCl_2_, 1 mM CaCl_2_, 2 mM KCl and 1% of bovine serum albumin) at room temperature. Subsequently, the slices (four to six slices per animal, 5 animals per group) were incubated with 1.4 nM of ^18^F-JNJ-64413739 in buffer solution during 30 min at room temperature. For the determination of non-specific binding, successive slices were incubated with the same radioligand solution containing 10 μM of non-labelled JNJ-64413739 (homologous blocking). After incubation, the slices were removed from the baths, washed twice in ice-cold buffer (50 mM Tris-HCl, pH 7.4, 4°C) and dipped once in ice-cold ultra-pure water. After drying over a heating plate (40°C), the slices were exposed to a phosphor sensitive plate for 5 min and the plate was scanned in a phosphor imager (Amersham Typhoon 5, GE, United States of America) at the highest resolution (10 μm). For image quantification, a region of interest was defined in the whole coronal brain slice with a specific software (ImageQuantTL, GE, United States of America) and a mean pixel intensity value was obtained for each slice. For each pair of slices, the percentage of blocking (specific binding) was calculated as [(No block- Block)/No block)] x 100. Statistical analysis (Nested *t*-test) was performed using Graphpad Prism (version 9).

### Statistics

The effects of JNJ-54175446 on spontaneous seizures were analysed with Stata Release 16.1. Ordinal logistic regression was used to test for the effect of treatment on seizure count within each of the three trial periods. Regression models included a term for day of trial and used robust variance estimation to account for clustering of observations within animals. Data was also separately graphed for data visualisation purposes. For other data, statistical analyses were performed using GraphPad Prism (version 9). Data were tested for normal distribution using D’Agostino and Pearson omnibus normality test or Shapiro-Wilk test for small n numbers. Parametric statistics used an unpaired two-tailed Student’s t-test (with Welch correction when assuming non-equal standard error of mean (SEM)) and data are presented as mean ± SEM. Non-parametric analyses used Mann-Whitney U test and data are presented as median ± interquartile range. For comparison of data with multiple parameters, a two-way repeated measures ANOVA was used. The individual tests used for each comparison are specified in the text. Data was considered significant at *p*-value ≤0.05.

## Results

### Spontaneous seizures in the IAKA model are refractory to levetiracetam

**FIGURE 1:**
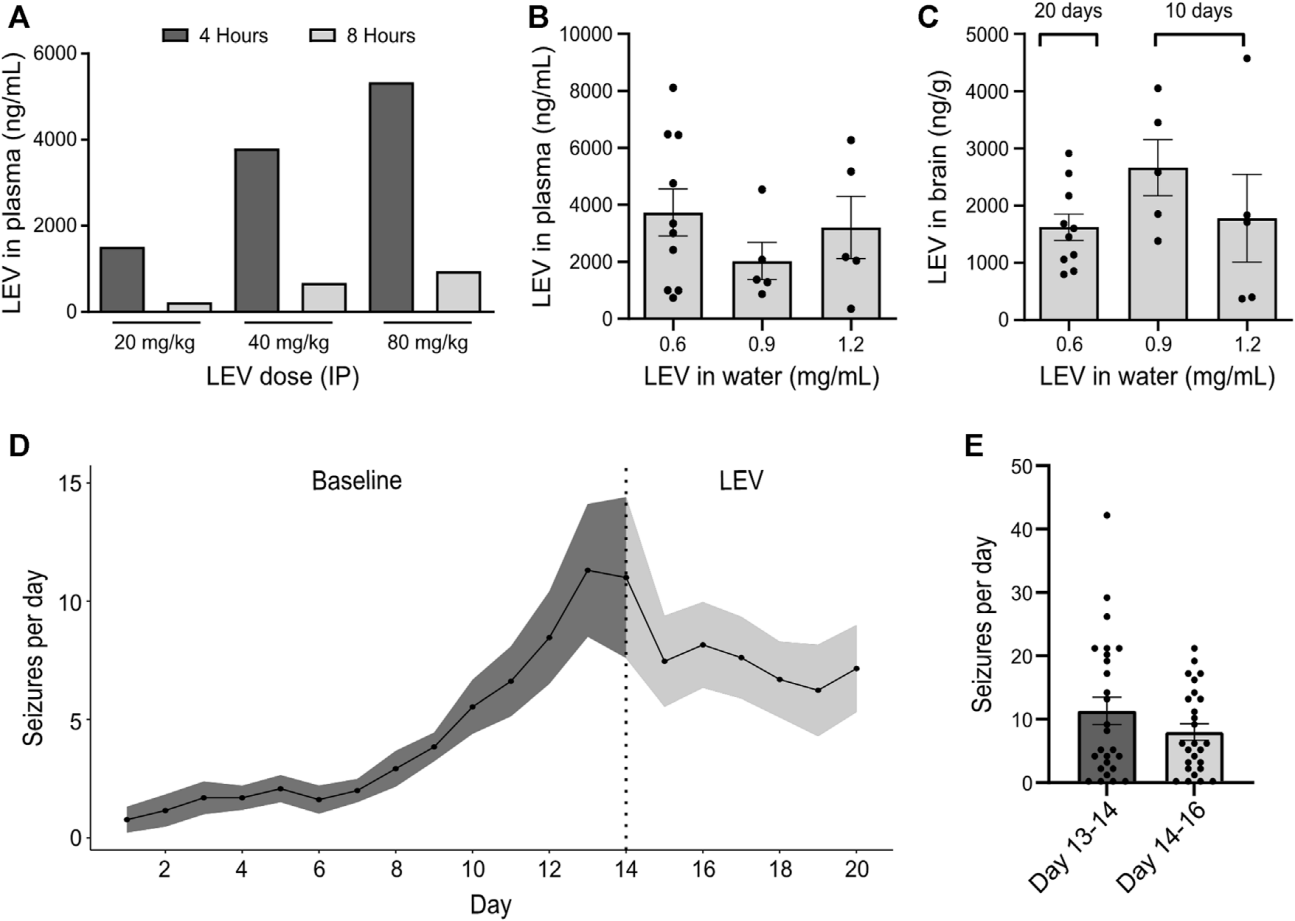
*Effect of levetiracetam in the IAKA model of* TLE **(A)** Dose range-finding pilot to gauge plasma levels of levetiracetam (LEV) 4 and 8 h following IP injection at various doses (n = 1/group). **(B)** Quantification of levetiracetam in plasma following 10 days of oral administration at different doses dissolved in water. Note that a dose of 0.6 mg/mL drinking water was sufficient to reach plasma levels comparable to those following an IP dose of 40 mg/kg (*p* = 0.437, one-way ANOVA, n = 5–10/group). **(C)** Summary of levetiracetam concentration in the brain following oral administration. Note that brain levels following a water concentration of 0.6 mg/mL was sufficient to reach brain levels of above 1000 ng/g, a concentration previously known to reduce seizure activity. **(D)** Spontaneous seizures following IAKA (n = 13). Note seizures continue after beginning levetiracetam treatment. **(E)** Graph comparing spontaneous seizure rates during the last 2 days of the baseline to the rates during the 2 days after treatment with levetiracetam. The change was not statistically significant (*p* = 0.191, unpaired *t*-test, seizure counts collected from n = 13 animals/day).

The pharmacological profile of the IAKA model has recently been explored and includes resistance to phenytoin and carbamazepine, and partial responses to phenobarbital, valproate and diazepam (West et al., 2022). Prior to testing JNJ-54175446, we sought to extend this characterisation by testing the response to levetiracetam, a common first-line drug to treat TLE (Kwan et al., 2011). Injection of 40 and 60 mg/kg levetiracetam (IP) resulted in plasma levels of 4000–5000 ng/mL at 4 h which declined to ∼1000 ng/mL by 8 h (Figure 1A). Next, mice were given levetiracetam dissolved in the drinking water and after 10 or 20 days, brain and plasma levels were determined. Dissolving 200 mg levetiracetam per 300 mL resulted in plasma and brain levels in mice similar to those achieved following IP dosing which are known to suppress seizures in various animal models (Figures 1B,C).

We next subjected a group of mice to IAKA. All mice developed status epilepticus and, within a few days, began displaying spontaneous seizures, consistent with the known profile in the model (Jimenez-Pacheco et al., 2016). Two weeks after IAKA when chronic epilepsy is established, the drinking water was switched to contain levetiracetam and rates of spontaneous seizures were followed for another week. Spontaneous seizures continued in mice after being switched to levetiracetam (Figure 1D). Rates were not statistically different when comparing 2 days before treatment to 2 days after (Figures 1D,E). These findings demonstrate that the spontaneous seizures in the IAKA model of TLE are largely refractory to levetiracetam.

### Plasma and brain levels of JNJ-54175446 after IP dosing

**FIGURE 2:**
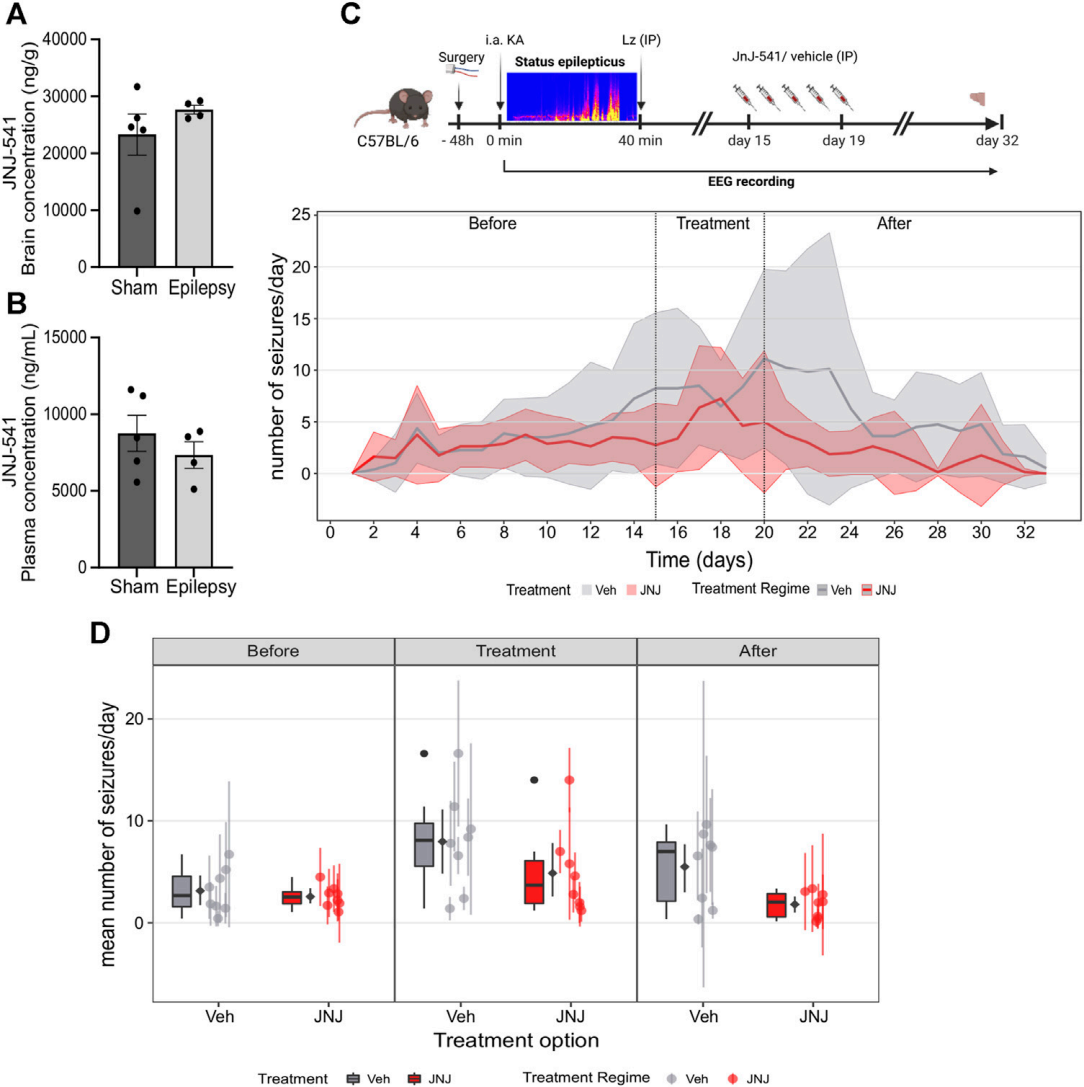
*Effect of JNJ-54175446 on spontaneous seizures in the IAKA model*. **(A)** Plasma levels of JNJ-54175446 (JNJ-541) measured 3 hours following a 30 mg/kg IP injection of the drug in naïve and IAKA animals (*p* = 0.387, unpaired *t*-test, n = 4–5/group). Levels exceed what has been shown to produce 80% receptor occupancy level. **(B)** Levels of JNJ-54175446 in the brain showed no difference (*p* = 0.299, unpaired *t*-test, n = 4–5/group) between naïve and IAKA animals following the same 30 mg/kg IP injection. **(C)** Schematic of experiment and (below) graph showing spontaneous seizures in the two groups. During JNJ-54175446 dosing there was a non-significant reduction in seizure frequency, but following drug washout, seizure frequency in JNJ-54175446-treated mice remained significantly lower than in vehicle-treated counterparts (n = 8/group). **(D)** Mean number of seizures for vehicle vs. JNJ-54175446-treated mice, showed a significantly lower seizure frequency in JNJ-54175446-treated mice following drug washout. Coloured scatters show animals median ± mad; diamond scatter indicates mean ± sd of mean values. Dark scatters show outliers excluded from mean, box and whiskers.

JNJ-54175446 was delivered via once-daily IP dosing at 30 mg/kg (Letavic et al., 2017). We first checked whether the presence of epilepsy would affect uptake in the brain, since the original pharmacokinetics studies were performed in healthy rodents (Letavic et al., 2017). Naïve mice and IAKA mice (2-week time-point) received a 30 mg/kg IP injection of JNJ-54175446. Brain and plasma samples were collected 3 hours after IP injection and processed for measurement of JNJ-54175446. Analysis revealed the IP injection of JNJ-54175446 (30 mg/kg) produced a high concentration of JNJ-54175446 in both the plasma and brain of control and epileptic mice, above what has been shown to produce 80% receptor occupancy level (Letavic et al., 2017). (Figures 2A,B). Thus, IP injection of JNJ-54175446 at 30 mg/kg to mice provides brain exposures that produce high levels of antagonism of the P2X7 receptor.

### JNJ-54175446 reduces spontaneous seizures in the IAKA model

To investigate the effect of JNJ-54175446 in the model, spontaneous seizures were recorded until day 14 after status epilepticus. Then, mice began to receive once-daily IP injections of JNJ-54175446 (30 mg/kg) or vehicle for 5 days. Dosing was then terminated and spontaneous seizures recorded for a further 13 days to allow drug wash-out, with mice killed on day 14.

Spontaneous recurrent seizures emerged within a few days of status epilepticus, consistent with the normal course of epilepsy in the model, and there was a small increase in seizure frequency over time between the first and second week (Figure 2C; Supplementary Figure S1A). At baseline, there were no differences in spontaneous recurrent seizures between the mice in the vehicle group and the mice that would go on to receive JNJ-54175446 (Figures 2C,D; Supplementary Figures S1A, B). During active dosing with JNJ-54175446, there was a small but non-significant reduction in spontaneous seizure rates in mice (Figures 2C,D; Supplementary Figures S1A, B). After dosing with JNJ-54175446/vehicle finished, spontaneous seizures continued to be monitored. Seizures in vehicle-treated mice continued at an average of ∼10 per day. In contrast, seizure rates in JNJ-54175446-treated mice remained low and there was a significant difference between JNJ-54175446-treated mice and vehicle-treated mice during the washout period (Figures 2C,D; Supplementary Figures S1A, B). That is, there was no difference in the seizure rate during the baseline phase (*p* = 0·858). In the treatment phase, the JNJ-54175446 group had a marginally lower overall seizure rate but this was not statistically significant (*p* = 0·096). However, in the post-treatment phase, the JNJ-54175446 group had a significantly lower seizure rate (*p* = 0·022). There was no effect of JNJ-54175446 on seizure durations during the dosing period or after dosing (Supplementary Figure S2).

### Effects of JNJ-54175446 on gliosis in the IAKA model

**FIGURE 3:**
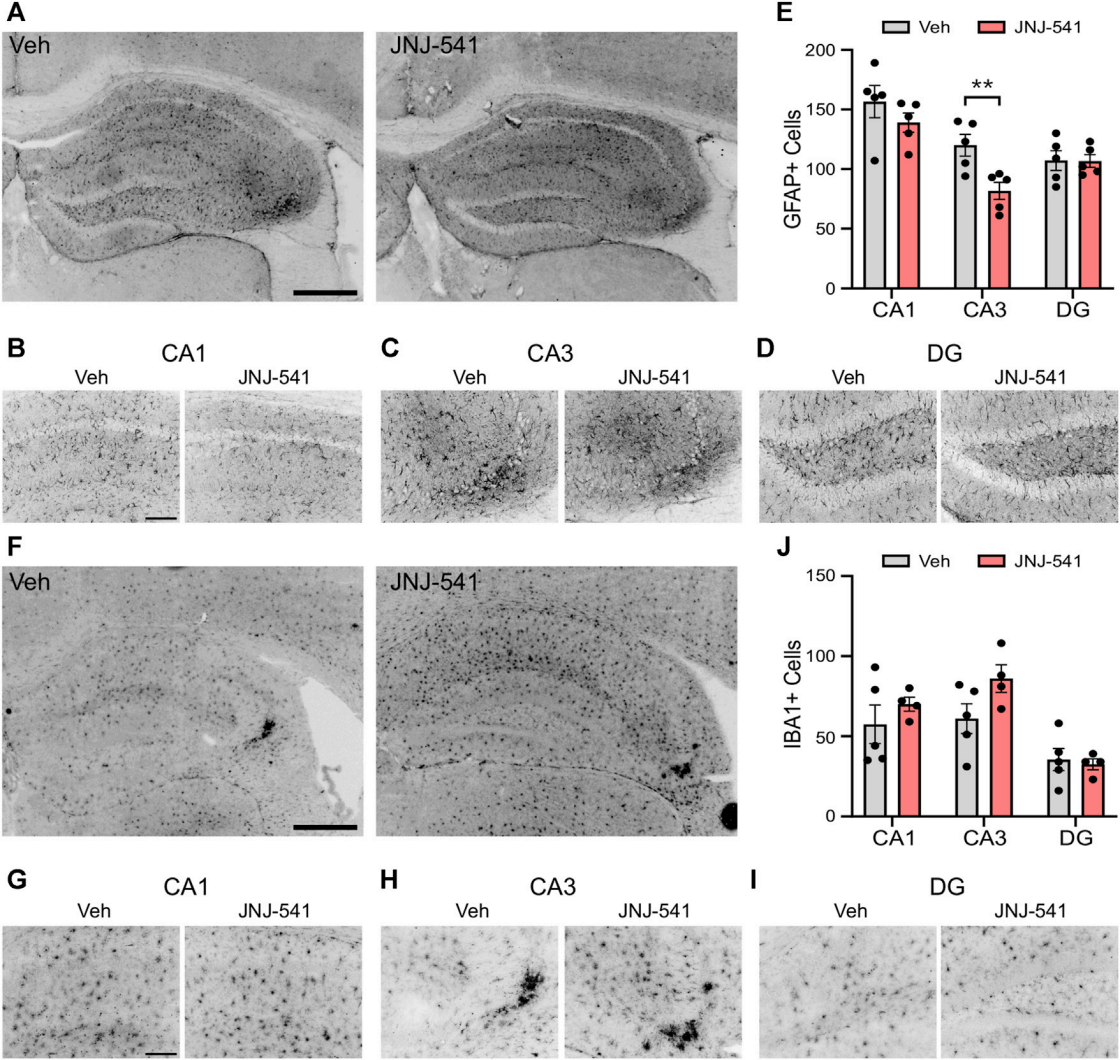
*Gliosis in mice treated with JNJ-54175446 in the IAKA model*. **(A–D)** Photomicrographs of representative GFAP staining in the hippocampus from vehicle and JNJ-54175446 (JNJ-541)-treated epileptic mice at the end of the experiment, showing ×5 field views (A) and ×20 lens magnifications **(B–D)** of individual subfields from the same animal. Note, lower numbers of GFAP + cells in the CA3 region of mice treated with JNJ-54175446 compared to mice treated with vehicle. Astrogliosis was similar in other subfields. **(E)** Quantification of GFAP + cells in each hippocampal subfield from vehicle and JNJ-54175446-treated mice, perfused 2 weeks after drug washout at the end of recordings (*p* = 0.034, two-way repeated measures ANOVA, n = 5/group). **(F–I)** Photomicrographs of representative IBA1 staining in the hippocampus from vehicle- and JNJ-54175446-treated epileptic mice, showing ×5 field views (F) and ×20 lens magnifications **(G–I)** of individual subfields from the same animal. Note similar staining between mice treated with vehicle as compared with JNJ-54175446. **(J)**, Quantification of IBA1+ cells found in each hippocampal subfield from vehicle- and JNJ-54175446-treated mice perfused 14 days after drug washout at the end of recordings (*p* = 0.219, mixed-effects analysis, n = 4–5/group). Scale bars: **(A,F)**, 500 μm; **(B–D)**, **(G–I)**, 100 μm.

Previous studies showed a 5-day treatment with JNJ-47965567 attenuates astrogliosis and microgliosis in the IAKA model (Jimenez-Pacheco et al., 2016). Accordingly, we analysed hippocampal sections from the vehicle and JNJ-54175446-treated mice at the end of recordings. Semi-quantitative analysis of astrogliosis in the hippocampus of vehicle control mice using GFAP immunostaining identified strong staining, particularly around the CA3 subfield ipsilateral to the site of IAKA (Figures 3A–E). GFAP staining was similar in brains from JNJ-54175446-treated mice in the CA1 and dentate gyrus, but was significantly reduced in the CA3 subfield (Figures 3A–E). Microgliosis was assessed by staining for IBA1. Strong staining for IBA1 was detected in the CA3 subfield of mice in both groups, as well as staining in both the CA1 and dentate gyrus but there were no differences in cell counts between vehicle- and JNJ-54175446-treated mice (Figures 3F–J). Consequently, we performed a further analysis of the morphology of IBA1-positive microglia processes, which may reflect differences in activation state (Paolicelli et al., 2022), in the CA3 subfield. This revealed IBA1-positive microglia in JNJ-54175446-treated mice displayed higher numbers of endpoints and longer process lengths compared to vehicle-treated mice (Supplementary Figure S3).

### *Ex vivo* autoradiography

**FIGURE 4:**
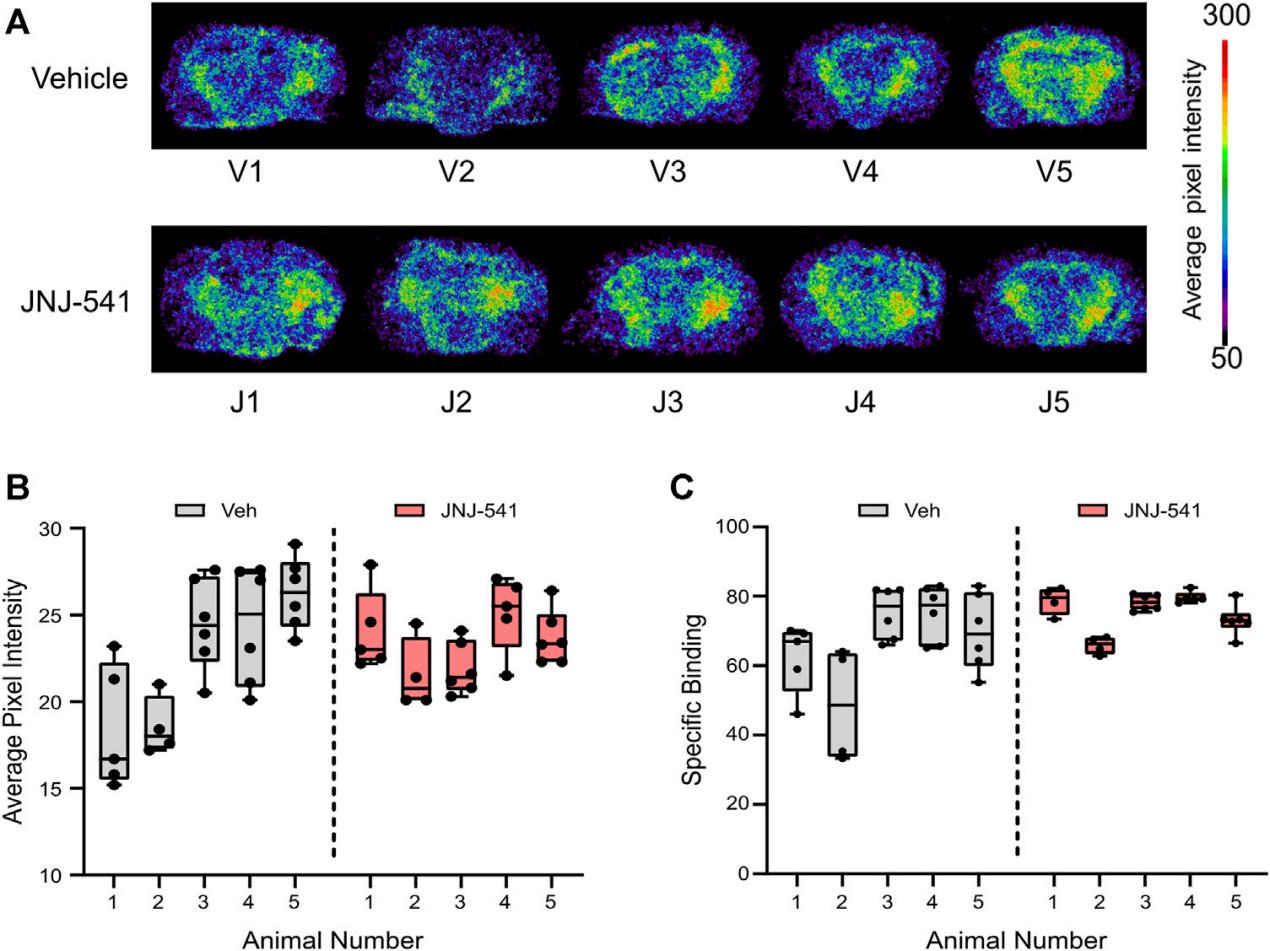
*Ex vivo P2X7 receptor radiotracer binding after long-term recording*s **(A)** Phosphor imaging of whole brain coronal slices showing localized average pixel intensity of bound P2X7 receptor radiotracer (n = 5/group). V1-V5 were treated with vehicle while J1-J5 were treated with the specific P2X7 receptor antagonist JNJ-54175446. **(B)** Average pixel intensity under non-blocking conditions of bound P2X7 receptor radiotracer on several whole brain slices of vehicle–and JNJ-54175446-treated epileptic mice. Nested *t*-test showed no statistically significant differences between groups. **(C)** Specific binding of P2X7 receptor radiotracer given as percentage of blocking ([(No block-block)/No block)] x 100).

Finally, to complement and extend the histologic findings, we sought to visualise P2X7 receptor levels in the brains of the mice. Here we employed an *ex vivo* radiotracer approach using ^18^F-JNJ-64413739 that we recently used to demonstrate enhanced P2X7 receptor levels in brain sections from patients with TLE (Morgan et al., 2023). The radiotracer ^18^F-JNJ-64413739 was applied to brain tissue sections from either vehicle or JNJ-54175446 groups and specific binding was confirmed under homologous blocking conditions (Figure 4A, Supplementary Figures S4A, S5). This revealed variable staining intensity between individual mice and groups. Semi-quantification of the tracer levels in whole brain sections revealed no statistical difference between groups in specific ^18^F-JNJ-64413739 labeling (Figures 4B,C). Similar results were obtained when the hippocampus and neocortex were separately analysed (Supplementary Figure S4B).

## Discussion

Attenuating neuroinflammation is a promising strategy for treatment-resistant epilepsies. Here we show that JNJ-54175446, a potent and selective antagonist of the P2X7 receptor, has anti-seizure effects in the IAKA model of treatment-resistant TLE in mice. We also extend the characterisation of the pharmacoresistance profile of the model, reporting the spontaneous seizures in the model are largely refractory to levetiracetam. Together, these findings support further investigation into the use of P2X7 receptor antagonists for the treatment of drug-resistant TLE and perhaps other epilepsies associated with a neuroinflammatory component.

Ongoing studies support neuroinflammation as a pathomechanism in treatment-resistant epilepsy and thus a target to achieve disease-modifying effects which are currently not provided by frontline anti-seizure medicines (Aronica et al., 2017; Beamer et al., 2017; Vezzani et al., 2019; Vezzani et al., 2022). This has led to recent clinical studies targeting the caspase-1/IL-1β system for treatment-resistant seizures, particularly where an inflammatory component is known or suspected (Lai et al., 2020; Yamanaka et al., 2021). The optimal approach to safe and protective modulation of neuroinflammation in epilepsy remains uncertain. Here, we tested an antagonist of the P2X7 receptor in a model of treatment-resistant TLE in mice. The P2X7 receptor represents a promising target since it lies upstream of the IL-1β pathway and is activated by high concentrations of extracellular ATP which likely occur mainly in the setting of tissue damage or excessive neuronal excitation (Beamer et al., 2017). This potentially avoids off-target effects of blocking the receptor outside the seizure focus or site of neuropathology (Beamer et al., 2017). The present study employed a potent, selective and brain-penetrant P2X7 receptor antagonist that has recently entered clinical trials (Letavic et al., 2017; Recourt et al., 2020; Recourt et al., 2023). The main finding here was that mice in the IAKA model treated with a short course of once-daily IP injections of JNJ-54175446 had fewer spontaneous seizures compared to vehicle-treated animals. This indicates that pharmacological blockade of the P2X7 receptor may be a suitable strategy for treatment-resistant epilepsy. If translated to a human trial setting, the observed three-fold lower seizure rates produced by JNJ-54175446 may represent a clinically-meaningful improvement in symptom control. Taking account of changes in the vehicle group, the main effect of JNJ-54175446 may have been to blunt the normal progression in seizure frequency in the model. Moreover, JNJ-54175446 was not superior to the previously tested P2X7 receptor antagonist JNJ-47965567 in the same (Jimenez-Pacheco et al., 2016). This raises the possibility that a ceiling has been reached with the amount of seizure suppression that can be achieved by targeting this receptor. Nevertheless, other properties including the requirement for less frequent dosing needed with JNJ-54175446 might influence decisions on selection for further development. A notable finding was that anti-seizure effects of JNJ-54175446 were most apparent at later time points, a delay also found in tests with JNJ-47965567 (Jimenez-Pacheco et al., 2016). As with the previous study (Jimenez-Pacheco et al., 2016), we did not observe an effect of P2X7 receptor inhibition on the duration of spontaneous seizures. Thus, antagonism of the P2X7 receptor appears to reduce the likelihood of a seizure occurring but does not influence the features of the event once a seizure is underway in this model. This contrasts with studies that found antagonism of the P2X7 receptor reduced the severity but not the frequency of spontaneous seizures in rats (Amhaoul et al., 2016). The explanation for the difference is unclear and may relate to the species, model, compound tested or dosing regimen. Regardless, the present study extends the evidence that targeting the P2X7 receptor can reduce seizures in experimental treatment-resistant TLE. Any preclinical development should take account of species-specific differences in the properties of rodent and human P2X7 receptors (Roger et al., 2010). In this regard, future studies could explore the present compound or subsequent iterations in other rodent or large animal models. For example, canines with naturally-occurring epilepsy would offer a potential translational model before moving to human studies (Loscher, 2022). Results with P2X7 receptor antagonists in other models (Fischer et al., 2016; Smith et al., 2023) suggest it may also be relevant to explore whether dosing with JNJ-54175466 immediately after status epilepticus has anti-epileptogenic effects in the model.

The mechanism of seizure suppression by JNJ-54175446 is unknown and was not explored presently. Since gliosis is an important driver of epileptogenesis and ictogenesis, the observed effects may derive from attenuation of gliosis. Indeed, both here and previously (Jimenez-Pacheco et al., 2016), we observed reductions in gliosis in mice after dosing with a P2X7 receptor antagonist. The model features select neuronal loss and gliosis, particularly within the ipsilateral CA3 subfield (Mouri et al., 2008; Jimenez-Pacheco et al., 2016), which is probably the site of ictogenesis (Li et al., 2008). Notably, this is the area where astrogliosis was reduced in the JNJ-54175446-treated mice. Moreover, the time-frame of the anti-seizure effects, emerging several days after dosing began, is slower than observed for conventional anti-seizure medicines in the model which have neuron-based mechanisms of action (West et al., 2022), and is more consistent with other cell type or higher-scale changes to network function. The reduction in astrogliosis may be secondary to reduced production of IL-1β, which is a potent trigger of astrogliosis (Allan et al., 2005; Vezzani et al., 2022). Here, JNJ-54175446 did not reduce microgliosis, as evidenced by microglia counts and indirectly via radiotracer studies for the P2X7 receptor. This was unexpected since microglia are the predominant cell type expressing the P2X7 receptor in mice (Kaczmarek-Hajek et al., 2018) and P2X7 receptor activation promotes microgliosis (Monif et al., 2009). We did, however, note a difference in the morphology of microglial processes which may be relevant to their influence on pathophysiology (Paolicelli et al., 2022). The finding contrasts the reduced microgliosis observed after dosing with JNJ-47965567 (Jimenez-Pacheco et al., 2016), and may be accounted for by a later end-point in the present study and waning of the anti-seizure effect re-provoking microgliosis. Non-glial-based mechanisms are also possible for the effects of JNJ-54175446 in the present study. While the function of neuron-based P2X7 receptor activity is uncertain (Armstrong et al., 2002; Sperlagh et al., 2002; Papp et al., 2004), studies have reported that the P2X7 receptor is over-expressed in neurons within a seizure focus (Rappold et al., 2006; Jimenez-Pacheco et al., 2016; Wei et al., 2016; Mikkelsen et al., 2023; Morgan et al., 2023). The delayed and net outcome of P2X7 receptor antagonism may therefore be a combination of effects of blocking the receptor on different cell types and longer-term changes secondary to resolving gliosis. This could be teased apart in future studies, for example, by studying the development and progression of epilepsy in mice lacking *P2rx7* in different cell types.

The IAKA model of TLE in mice displays a drug-resistance profile suitable to identify novel anti-seizure and disease-modifying therapies (Welzel et al., 2020; West et al., 2022). Previously, high-dose levetiracetam was shown to reduce the number of spontaneous seizure-like events and block generalized convulsive seizures in the intrahippocampal kainate model in mice (Klein et al., 2015). Here, we found that the spontaneous seizures in the intraamygdala kainic acid model are largely resistant to levetiracetam, extending work which showed resistance to both phenytoin and carbamazepine and partial responses to diazepam, phenobarbital and valproate (West et al., 2022). The finding is important, therefore, since levetiracetam has a different mechanism of action to these, acting via modulation of SV2A and neurotransmitter release rather than ion channels and neurotransmitter receptors. Nevertheless, levetiracetam, as with the other compounds, has a neuro-centric mechanism of action. Since gliosis is a critical pro-epileptogenic stimuli (Ortinski et al., 2010; Robel et al., 2015), compounds which attenuate gliosis such as P2X7 receptor antagonists, are important additions to the drug armamentarium. Targeting the P2X7 receptor may have applications in other focal epilepsies. That is, non-TLE forms of treatment-resistant epilepsy also associated with gliosis and neuroinflammatory signaling (Wei et al., 2016; Iffland et al., 2017).

There are a number of limitations to consider in the present study. We did not perform combined video-EEG monitoring which could have provided information on whether the drug mitigates behavioral components of spontaneous seizures. We did not explore whether JNJ-54175446 affected levels of the P2X7 receptor at earlier time-points or prove the anti-seizure effects are specifically through targeting the P2X7 receptor, for example, via attenuation of IL-1β responses. We may not have achieved optimal dosing with JNJ-54175446. While the pharmacokinetics of JNJ-54175446 suggest that a once-daily IP injection of 30 mg/kg should achieve sufficient receptor occupancy in the brain, different routes, earlier introduction of the drug relative to the inciting status epilepticus, repeating administration or higher doses may have produced greater or more lasting suppression of seizures. Although we have asserted the model is refractory to levetiracetam, the drug appeared to blunt the rising number of spontaneous seizures. We expect to have achieved a sufficient brain level of levetiracetam in our studies but cannot exclude that higher doses or delivery via another route would have produced different results. Finally, it is unclear whether antagonism of the P2X7 receptor alone will be sufficient to achieve complete seizure suppression, even when dosing is fully optimized. It would be interesting, therefore, to explore whether a period of treatment with JNJ-54175446 or another P2X7 receptor antagonist would enhance the effects of a conventional anti-seizure medicine such as levetiracetam. Indeed, studies suggest that a combinatorial approach blocking P2X7 receptor activity may render mice more susceptible to the anti-convulsant effects of benzodiazepines in models of status epilepticus (Beamer et al., 2022). Such a combination would also hold translational value since any future clinical trial of a P2X7 receptor antagonist would no doubt require add-on dosing with an existing anti-seizure medicine (Simonato et al., 2012).

In summary, the present study supports the targeting of the P2X7 receptor for the treatment of drug-resistant TLE and extends the evidence for the pharmacoresistance profile of the IAKA model in mice. Future studies could explore optimal dosing, perhaps in combination with a conventional anti-seizure medicine, and explore the cell type-specific effects of antagonism of the receptor.

## Data Availability

The original contributions presented in the study are included in the article/Supplementary Material, further inquiries can be directed to the corresponding author.
