# Supplementary material for: Anti-seizure effects of JNJ-54175446 in the intra-amygdala kainic acid model of drug-resistant temporal lobe epilepsy in mice
Source: Front Pharmacol. 2024 Jan 8;14:1308478. doi: 10.3389/fphar.2023.1308478 (PMC10800975; doi:10.3389/fphar.2023.1308478)
Supplement: Supplementary file 1 [file DataSheet1.PDF]

Anti-seizure effects of JNJ-54175446 in the intra-amygdala kainic acid model of drug-resistant temporal lobe epilepsy in mice

Omar Mamad, Mona Heiland, Andreas Lindner, Thomas D. M. Hill, Ronan M. Ronroy, Kilian Rentrup, Amaya Sanz-Rodriguez, Elena Langa, Janosch P. Heller, Oscar Moreno, Jordi Llop, Anindya Bhattacharya, James A. Palmer, Marc Ceusters, Tobias Engel and David C. Henshall

Supplementary Figures 1 - 5

Supplementary Data Figure S1

A

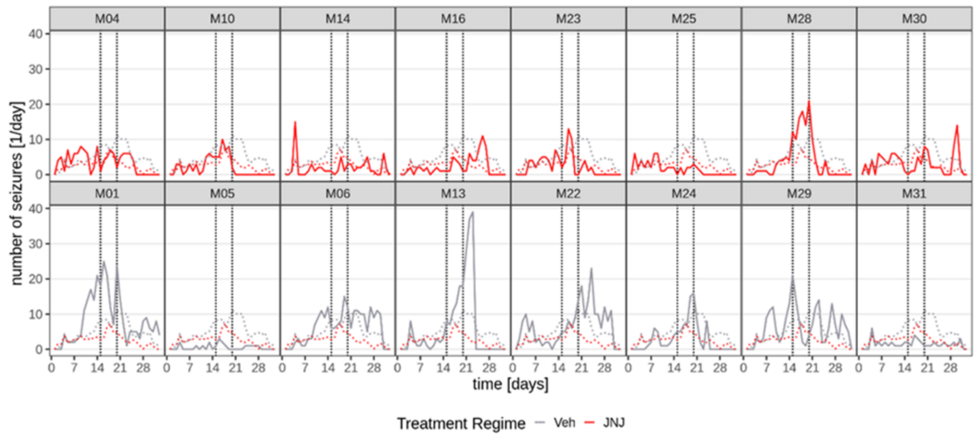

B

Period	Odds ratio	95% CI			z	P
Pre-treatment	1.1	0.362	–	3.387	0.2	0.858
Treatment	0.18	0.023	–	1.364	-1.7	0.096
Post-treatment	0.08	0.009	–	0.691	-2.3	0.022

Individual animal data during dosing with JNJ-54175446

(A) Individual animal results before, during and after dosing with JNJ-541 or placebo. The left vertical bars mark the start of JNJ-541/vehicle dosing, the right bars the end of dosing.

(B) Statistical analysis. At baseline, there were no differences in spontaneous recurrent seizures between the mice in the placebo group and the mice that received JNJ-541. During active dosing with JNJ-541 there was a small but non-significant reduction in spontaneous seizure rates in mice. After dosing with JNJ-541 finished, spontaneous seizures continued to be monitored. Seizures in placebo mice continue at an average of ~10 per day. In contrast, seizure rates in JNJ-541-treated mice remained low and there was a significant difference between JNJ-541 and placebo during the washout period. That is, there was no significant difference in the seizure rate over the pre-treatment phase ($p = 0.858$). In the treatment phase, the JNJ-541 group had a marginally lower overall seizure rate but this was not statistically significant ($p = 0.096$). However, in the post-treatment phase, the JNJ-541 group had a significantly lower seizure rate, with an odds ratio of 0.08 ($p = 0.022$).

Supplementary Data Figure S2

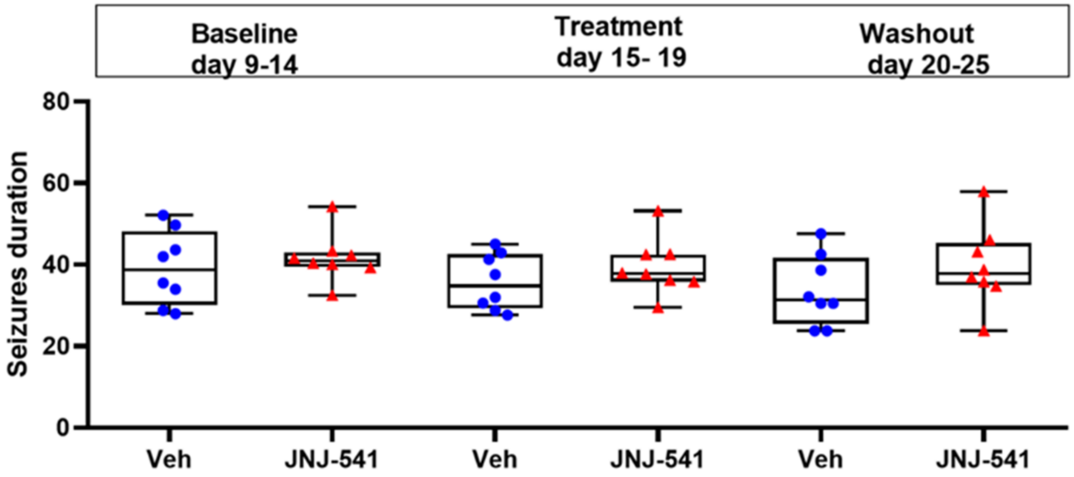

Effect of JNJ-54175446 on the duration of spontaneous recurrent seizures in mice

Graph shows averaged data for spontaneous seizure duration (in seconds) in mice for vehicle- and JNJ-541-treated mice during the three phases of the study. Note seizure duration remained similar throughout with no difference between groups. N = 8/group.

Supplementary Data Figure S3

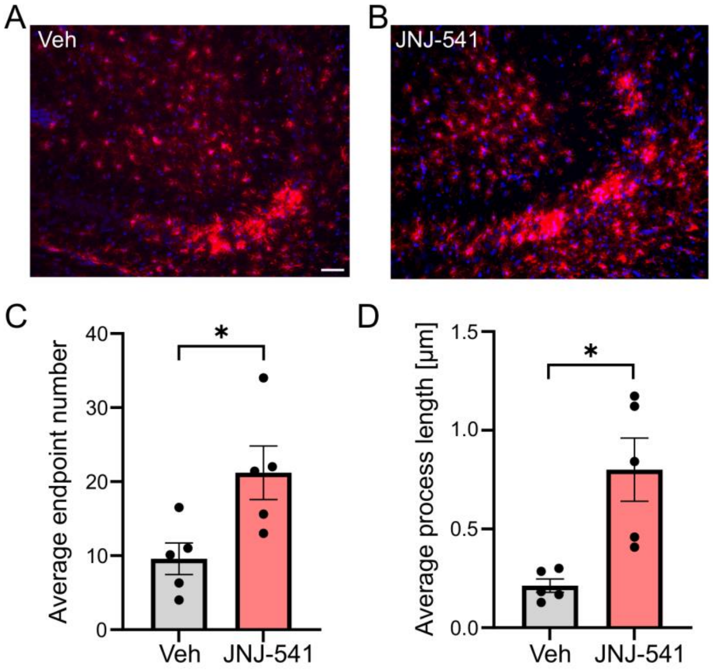

Microglia phenotypes in mice treated with JNJ-54175446 in the IAKA model.

(A-B) Photomicrographs of representative IBA1 staining in the hippocampus from vehicle and JNJ-54175446 (JNJ-541)-treated epileptic mice. (C) The number of endpoints was significantly higher in JNJ-541-treated epileptic mice ($p = 0.0248$, unpaired t-test, $n = 5$/group). (D) The average process length was significantly longer in JNJ-541-treated epileptic mice ($p = 0.0200$, unpaired t-test, $n = 5$/group). Scale bar: A, 100 μm.

Supplementary Data Figure S4

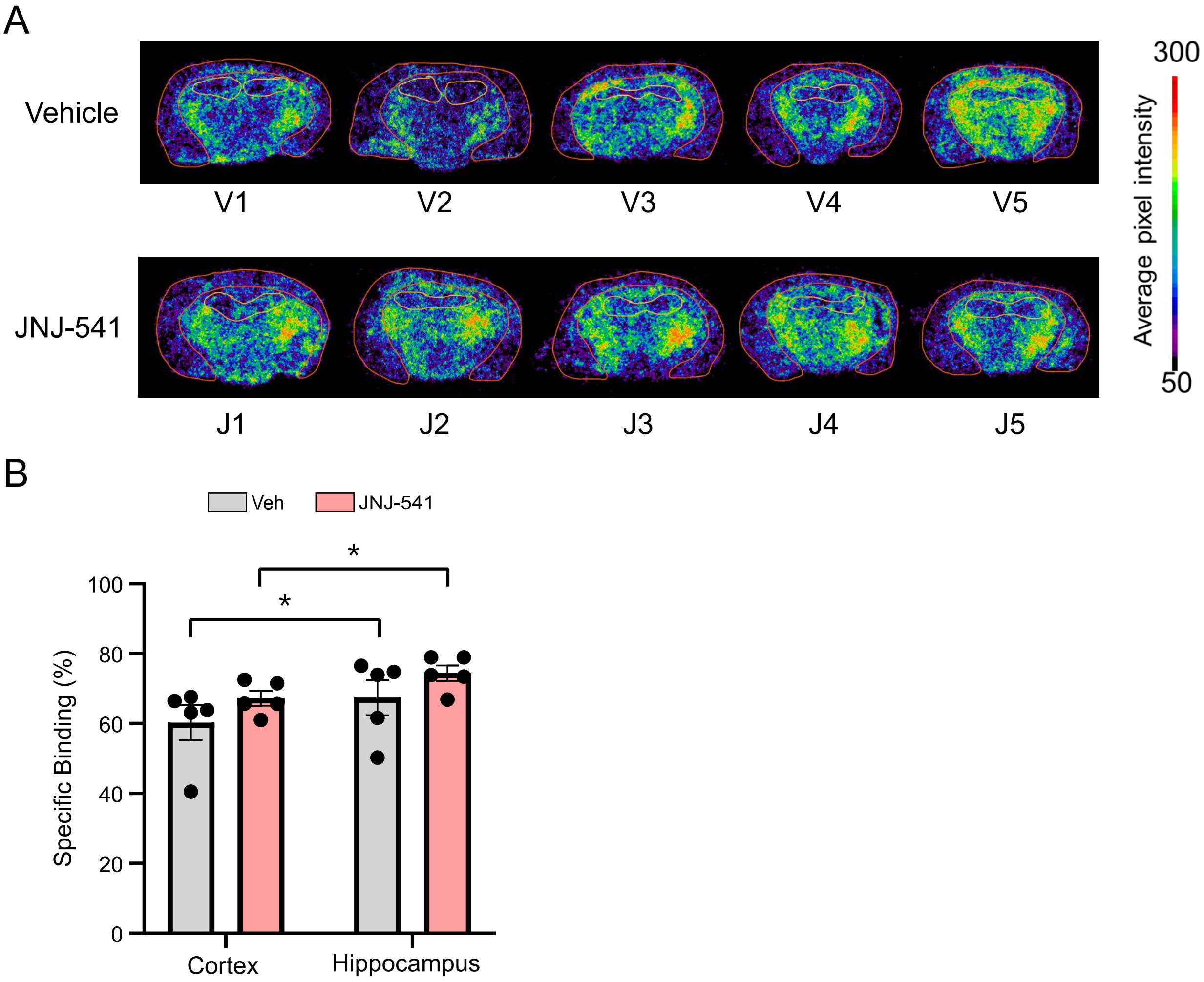

Ex vivo P2X7 receptor radiotracer binding in sub-regions of interest

(A) Phosphor imaging of whole brain, sub-regions of interest (hippocampus and cortex) are drown in red color showing localized average pixel intensity of bound P2X7 receptor radiotracer ^{18}F-JNJ-64413739. V1-V5 were treated with vehicle while J1-J5 were treated with the specific P2X7 receptor inhibitor JNJ-54175446 (JNJ-541). (B) Specific binding of the P2X7 receptor tracer in cortex and hippocampus given as percentage of blocking ([(No block- Block)/No block)] x 100) (Vehicle cortex vs. hippocampus: $p = 0.0197$, JNJ-541 cortex vs. hippocampus: $p = 0.0193$, two-way repeated-measure ANOVA, n = 5/group).

Supplementary Data Figure S5

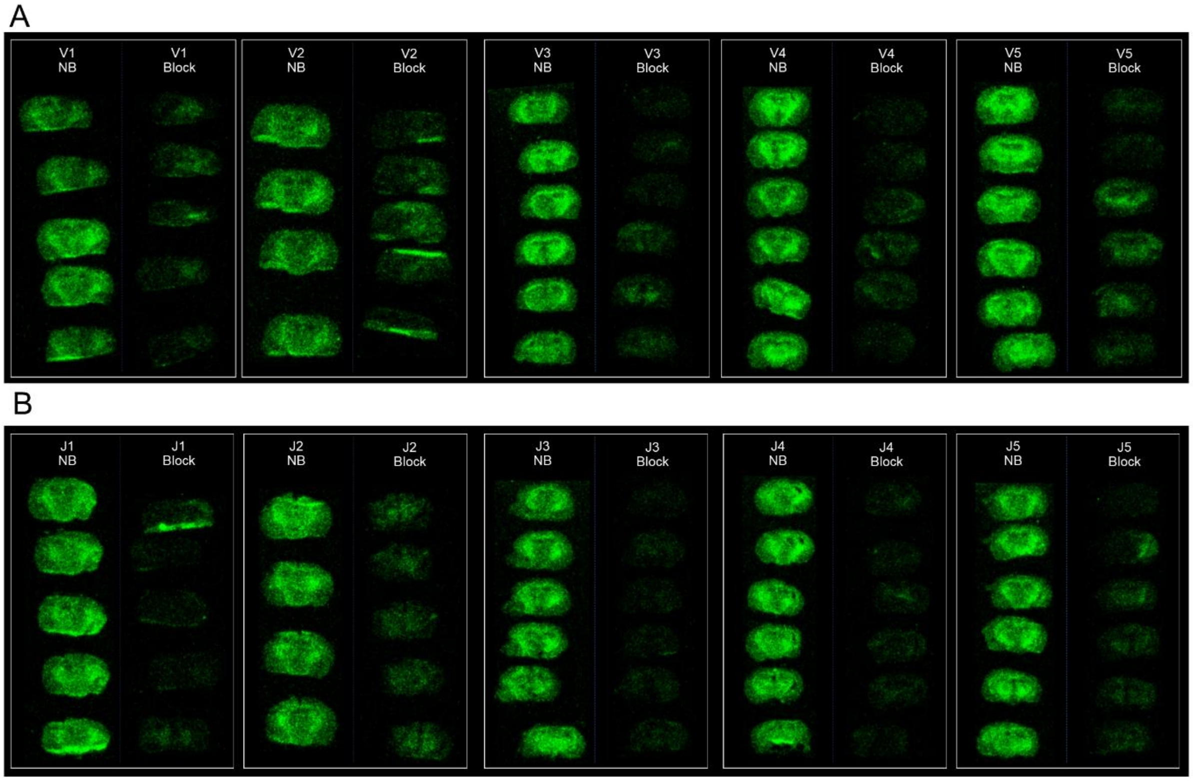

Ex vivo P2X7 receptor radiotracer binding after long-term recordings

(A-B) Imaging of P2X7 receptor radiotracer ^{18}F-JNJ-64413739 binding expression in bound and non-bound states for vehicle-treated (A) and JNJ-54175446-treated (B) epileptic mice. N = 5/group.
